# Effect of a Novel Ergonomic Sheath on Dental Device-Related Muscle Work, Fatigue and Comfort—A Pilot Clinical Study

**DOI:** 10.3390/dj12090296

**Published:** 2024-09-21

**Authors:** Steven Dang, Cherie Wink, Susan Meishan Yang, Kairong Lin, Thair Takesh, Ali A. Habib, Petra Wilder-Smith

**Affiliations:** 1 Beckman Laser Institute, Department of Surgery, University of California Irvine School of Medicine, Irvine, CA 92612, USA; stevenld@uci.edu (S.D.); cwink@hs.uci.edu (C.W.); susanmy@uci.edu (S.M.Y.); kaironl@uci.edu (K.L.); ttakesh@uci.edu (T.T.); 2 Department of Neurology, University of California Irvine School of Medicine, Orange, CA 92868, USA; aahabib@hs.uci.edu

**Keywords:** ergonomic sheath, dental hygienist, dentist, ergonomics, ultrasonic scaler, micromotor, electromyography, musculoskeletal disorder

## Abstract

**Background:** Dental instrumentation with hand-held devices is associated with discomfort, fatigue and musculoskeletal diseases or repetitive stress injuries. The goal of this in vivo study was to determine the effect of an ergonomic handle sheath on muscle work, comfort and fatigue associated with (a) piezoelectric scaling by hygienists with and without musculoskeletal disorders (MSDs), and (b) dental cavity preparation by healthy dentists using a dental micromotor. **Materials and Methods:** Two groups of ten hygienists each tested the piezoelectric scaler. Hygienists in Group 1 had no MSDs, while those in Group 2 had been diagnosed with MSDs. Additionally, ten dentists with no MSDs used a dental micromotor to prepare four standardized cavities. Time-based work in four muscles, comfort and fatigue were recorded in the presence and absence of an add-on soft, insulating handle sheath. Data were analyzed using a repeated measures analysis of variance model with Tukey’s post-hoc test. **Results:** Comfort, fatigue and muscle work were significantly better for both devices when the sheath was used. While hygienists with MSDs used more muscle work to complete the set scaling task, and the sheath-related reduction in work was somewhat greater, these MSD-related differences did not quite reach significance. **Conclusions:** The results of this pilot study show that the ergonomic performance of an ultrasonic scaler and a dental micromotor may be improved by the use of an ergonomic handle sheath.

## 1. Introduction

Given the alarming prevalence and severity of instrumentation-related musculoskeletal disorders (MSDs) in dental hygienists and dentists, novel approaches to the prevention and mitigation of such disorders and injuries are urgently needed. Work-related disorders such as MSDs and chronic inflammatory conditions are reported to occur in 54–93% of dentists and dental hygienists [1]. These injuries are most common in dental hygienists [2]. They tend to be extensive and long-lasting and typically involve body sites such as the vertebral column, shoulder, wrist, hand and fingers [1,2,3,4,5,6,7,8,9,10,11,12]. Indeed, many hygienists are compelled to reduce their working hours after just a few years in clinical practice due to the limitations resulting from their work-related MSDs [10,13,14]. The situation for dentists is not much better, with the majority of them reporting musculoskeletal pain from the repetitive, high-loading activities that are associated with dental practice [12,13,14,15,16,17,18,19,20,21,22,23,24,25,26,27,28,29,30]. MSD-related pain, poor sleep quality, reduced work satisfaction and earnings losses were found to affect almost one-half of all dentists [31]. More than one-quarter of all dentists retire early due to work-related pain and disability [15], with adverse personal and financial consequences. The financial cost of these work-related injuries is considerable: In one study, investigators estimated an annual income loss of $131 million due to MSDs in the dental profession [20,32].

A wide range of new dental materials with more beneficial properties are under development. Dental hand instruments that have typically been manufactured as linear, elongated, rigid metal tools are now being re-designed with softer, wider, lighter non-metal handles to avoid the poor musculoskeletal properties of thin, linear, hard, cold handles. This is because extensive studies have determined that somewhat larger diameter, non-linear, lighter tools with silicone-covered, warmer and softer instrument handles inflict less musculoskeletal stress on the finger-hand-arm apparatus [33,34,35,36,37,38]. Revised instrumentation techniques to prevent and mitigate pain have also been researched [32]. Moreover, new methodologies for tracking finger, hand, arm and finger movements during instrumentation, as well as novel surface Electromyography (sEMG) techniques now permit accurate, real-time mapping of the effects of instrument materials and design on instrument efficacy, as well as the clinician’s technique, positioning and muscle work. However, there have been fewer improvements in the handle design of power-driven dental tools.

It is surprising to note that, despite the extensive literature documenting the presence, prevalence and implications of MSDs in dental clinicians, little has been published about the effect of these MSDs on the amount of muscle work required to complete a set instrumentation task, and the associated discomfort and fatigue. However, in one recent pilot study, researchers investigated the effects of MDSs in relation to the work required to complete a standardized scaling task using periodontal curettes [39]. Investigators reported that individuals with MSDs experienced approximately 70% worse comfort, 100% more fatigue and used more than twice the amount of muscle work than their healthy counterparts to complete their instrumentation task [39]. These findings provide a strong impetus for additional research on this topic as a means of finding new and better ways of addressing the challenge of MSDs in dental clinicians.

The goal of this in vivo study was to determine the effect of an ergonomic handle sheath on muscle work, comfort and fatigue associated with (a) piezoelectric scaling by hygienists with and without MSDs, and (b) dental cavity preparation by healthy dentists using a dental micromotor. 

## 2. Materials and Methods

After review by the University of California, Irvine’s Internal Review Board IRB, this protocol was granted exempt status, as only de-identified data were collected and used in this study. All testers were informed of their right to withdraw from the study at any time and for any stated or unstated reason.

All testing was performed by dental hygienists and dentists using typodont models mounted in dental manikins and attached to a standard clinical dental chair. All clinicians performed the same testing protocol twice: once with the ergonomic sheath in place, and once without, with the sequence of use randomized on a 1:1 basis using the research software randomiser.org (last accessed 23 August 2023). The two study arms were separated by a 10-min resting period. Return to baseline muscle activity at the end of the resting period was confirmed by sEMG. All testers wore gloves during testing (Dental City Stratus Nitrile Powder Free Gloves, Green Bay, WI, USA). 

### 2.1. Ergonomic Sheath (Figure 1)

**Figure 1:**
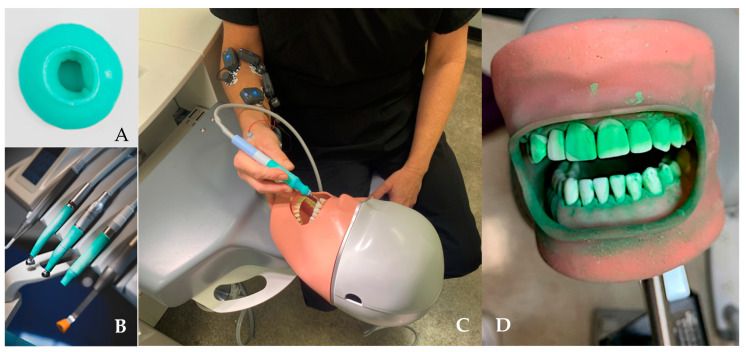
(**A**) The ergonomic sheath is provided as a rolled-up ball, which is placed on the instrument end and unrolled to apply to the grip area (**B**). (**C**) Clinician with sEMG electrodes in place using scaler with ergonomic sheath (green) in place. (**D**) Typodont with artificial calculus and biofilm.

The ergonomic sheath (Figure 1) used in this study is made of medical-grade silicon (Handix, Oslo, Norway). It was applied to the dental handpieces and piezoelectric scalers by simply rolling it onto the dental device handle. The sheath is approximately 1 mm thick and is designed to provide thermal insulation as well as a mild cushioning effect for the fingers gripping the device. The durometer of the sheath material was specifically constituted to dampen the vibrational forces generated while maintaining excellent tactile feedback for the clinician.

### 2.2. (a) Ultrasonic Scaling—Testers

Twenty right-handed hygienists participated in this study. Group 1 consisted of ten testers who had experienced no injuries or disorders of their fingers, hands or wrists within 6 months of study begin, and who had neither symptoms nor a diagnosis of upper-extremity MSDs. Testers in Group 2 had been diagnosed within 3 months of this study with chronic MSDs in hands, fingers, arms and/or shoulders by their medical care providers and were unable to work full-time as clinicians due to related pain and disability. 

### 2.3. (b) Ultrasonic Scaling—Protocol

Artificial biofilm (Occlude Green Marking Spray, Pascal International. Bellevue, WA, USA) and calculus (Dental Calculus Set, Kilgore International Inc., Coldwater, USA) were applied supra- and sub-gingivally in a standardized fashion to the 32 artificial typodont teeth that were subsequently mounted in each typodont (Kilgore International Inc., Coldwater, MI, USA) (Figure 1). The artificial biofilm and calculus were applied 18 h before scaling took place, as artificial calculus increases in hardness over time. Next, the typodont was mounted in a standard dental manikin, which was in turn attached to a clinical dental chair. The hygienists were seated in a conventional position at the dental chair. They were allowed to change their seating position and adjust the manikin position as needed throughout the study. A researcher observed each tester throughout the study and noted whenever an adjustment of this kind was made so that the resultant disruptions in the sEMG trace could be identified.

Hygienists all used a Woodpecker ultrasonic piezoelectric scaler (Gulin, China). This scaler’s handle has a hard, non-metal surface. Testers were instructed to complete periodontal scaling as if they were working on a live patient, with the goal of removing the simulated plaque and calculus deposits completely without damaging the underlying teeth or simulated soft tissues. Testers all followed the same scaling routine: (1) lower anterior sextant facial surfaces, (2) lower anterior sextant lingual surfaces, (3) upper anterior sextant facial surfaces, (4) upper anterior sextant lingual surfaces, (5) lower right sextant buccal surfaces, (6) lower left sextant buccal surfaces, (7) upper right sextant buccal surfaces, and (8) upper right sextant lingual surfaces. Testers did not perform a full mouth scaling as some of the hygienists with MSDs said this would be too taxing for them.

### 2.4. (a) Cavity Preparation Using a Dental Micromotor—Testers

Ten right-handed dentists, who are all experienced clinicians with more than 5 years of clinical experience, participated in this study. They had experienced no injuries or disorders of their fingers, hands or wrists within 6 months of study beginning, and had neither symptoms nor a diagnosis of upper-extremity MSDs. 

### 2.5. (b) Cavity Preparation Using a Dental Micromotor—Protocol

Extracted teeth were mounted in a typodont model, which was then attached to a manikin (Kilgore International Inc., Coldwater, MI, USA), which was in turn attached to a clinical dental chair. The dentists were seated in a conventional position at the dental chair. They were allowed to change their seating position and adjust the manikin position as needed throughout the study. A researcher observed each tester throughout the study and noted whenever an adjustment of this kind was made so that the resultant disruptions in the sEMG trace could be identified. Each tester prepared one Class V filling in the upper left central incisor and another Class V filling in the lower right canine, as well as one Class II 3-surface filling in the upper right first molar and one Class II 3-surface posterior filling in the lower left second molar. A KaVo steel micromotor (INTRA LUX KL703 LED, KaVo Dental Technologies LLC., Charlotte, NC, USA) was used to prepare the cavities. This micromotor features a hard metal handle. 

### 2.6. Surface Electromyography (sEMG)

Real-time, continuous surface EMG (sEMG) measurements were recorded in each clinician using surface EMG electrodes (FREEEMG, ©BTS Engineering, Quincy, MA, USA). The sEMG electrodes were affixed by the same dental clinician (CW) each time to ensure measurement parity across testers. The adhesive, disposable electrode pads were affixed directly over 4 muscles (Figure 1) that are specifically used for gripping and manipulating dental devices [2,5,33,35,36,40,41,42,43]: the Abductor Pollicis Brevis (APB), First Dorsal Interosseous (FDI), Flexor Pollicis Longus (FPL) and Extensor Digitorum Communis (EDC). The sEMG mapping was performed using a standardized sequence [44] that (1) ensured accurate and optimal electrode placement [45], (2) established baseline maximum voluntary contraction (MVC) values over 15 s for each muscle to allow for subsequent data normalization [46,47,48,49] and (3) mapped muscle work throughout instrumentation. Surface EMG (sEMG) signals from all four muscles were recorded throughout instrumentation. After data collection was completed, raw sEMG signals were rectified and filtered using a second-order Butterworth filter with a 10 Hz high pass cutoff frequency using the BTS EMG analyzer^TM^ software (version 1, FREEEMG, ©BTS Engineering, Quincy, MA, USA). Finally, total muscle activity was calculated from the integrated EMG curve, which measures the total area under the curve (total workload) over the entire period of instrumentation.

### 2.7. VAS Surveys and Open-Ended Comments

VAS surveys using a hard-copy scale of 0–10 were completed immediately at the end of each study arm. One survey evaluated tester fatigue, and the second documented user comfort in wrist, fingers and palm, with 0 being no fatigue or discomfort and 10 being extreme fatigue or discomfort. Finally, participants were asked to provide written comments assessing the performance of the ergonomic handle sheath. 

### 2.8. Statistical Analysis

Standard SPSS 19 statistics software (IBM^®^, Armonk, NY, USA) was used to perform data analysis of the sEMG data by means of a repeated measures analysis of variance model with Tukey’s post-hoc test. The level of significance was set at *p* < 0.05.

## 3. Results

All testers were right-handed and completed the study in full compliance with the protocol. The ten hygienists without MSDs were all female, ranged in age from 24–56 years (mean age 36.1 years) and had 3–30 years of clinical experience (mean 16 years). The hygienists with MSDs were also all female, ranged in age from 47–68 years (mean age 49.3 years) and had 10–32 years of clinical experience (mean 18 years). The age difference between the two tester groups was significant (*p* = 0.0092). The difference in years of experience did not reach statistical significance (*p* = 0.0584). The dentists without MSDs who participated in this study ranged from 31–49 years of age (mean 42.7 years) and had 9–26 years of experience (mean 19 years). Six were female and four were male. 

### 3.1. (a) Surface EMG Data—Scaling (Table 1)

Hygienists with MSDs expended more work to complete the set scaling task without using the ergonomic sheath than their healthy colleagues, but the difference did not reach significance (*p* = 0.4309) (Table 1).

All hygienists (without and with MSDs) expended significantly less total work (*p* = 0.0079 and *p* = 0.0028, respectively) to complete a full scaling with the ergonomic sheath than without it. The sheath-associated reduction in work was slightly greater in the group of testers with MSDs, but this difference was not statistically significant (Table 1).

**Table 1:** Mean total muscle work expended by 10 hygienists to complete scaling.

	No MSD, No Sheath, (n = 10)	No MSD, Sheath (n = 10)	MSD, No Sheath (n = 10)	MSD, Sheath (n = 10)
Mean total muscle work (mV)	0.704	0.539	0.746	0.577
Std. Deviation	0.118	0.167	0.115	0.180

### 3.2. (b) Surface EMG Data—Cavity Preparation (Table 2)

Dentists performed significantly less total work with the sheath than without it during preparation of two standard cavities in anterior (*p* = 0.0154) and posterior (*p* = 0.001) teeth, respectively. Using the sheath significantly reduced total muscle work in both intraoral locations (Table 2).

**Table 2:** Mean total muscle work expended by 10 dentists without MSDs.

	No Sheath: Anterior Teeth (n = 10)	Sheath: Anterior Teeth (n = 10)	No Sheath: Posterior Teeth (n = 10)	Sheath: Posterior Teeth (n = 10)
Mean	0.968	0.713	1.334	1.081
Std. Deviation	0.114	0.286	0.089	0.166

### 3.3. (a) Comfort and Fatigue—Scaling (Table 3)

Without the sheath, all comfort and fatigue parameters were significantly better (*p* < 0.0001) in healthy testers than in those with MSDs. When the hygienists with MSDs used the sheath, their comfort and fatigue levels were closer to those of the healthy group, with only the finger comfort (*p* < 0.0001) and fatigue (*p* = 0.002) categories remaining significantly worse in this group vs. the healthy group (Table 3).

In testers without MSDs, when the sheath was used, mean comfort improved significantly in the palm (*p* = 0.0051) and the wrist (*p* = 0.015), but not in the fingers (*p* = 0.081). Mean fatigue was also significantly less in healthy testers when the sheath was used (*p* = 0.0002) (Table 3). In clinicians with MSDs, mean comfort improved significantly in the palm (*p* < 0.0001), wrist (*p* < 0.0001) and fingers (*p* < 0.0001). Mean fatigue was also significantly less in testers with MSDs when the sheath was used (*p* < 0.0001) (Table 3).

All parameters in both tester groups were significantly better when the sheath was used, except for comfort in the fingers in healthy testers.

**Table 3:** Mean Comfort and Fatigue in hygienists during scaling on a scale of 0–10, where 0 is best and 10 is worst.

	No MSD, No Sheath, (n = 10)	No MSD, Sheath (n = 10)	MSD, No Sheath (n = 10)	MSD, Sheath (n = 10)
Mean Comfort Palm (S.D.)	3.2 (0.33)	2.6 (0.299)	5.7 (0.523)	3.1 (0.338)
Mean Comfort Wrist (S.D.)	2.4 (0.216)	1.9 (0.168)	4.8 (0.529)	2.7 (0.312)
Mean Comfort Fingers (S.D.)	1.7 (0.183)	1.4 (0.116)	5.3 (0.449)	2.8 (0.297)
Mean Fatigue (S.D.)	1.9 (0.238)	1.1 (0.138)	6.0 (0.555)	3.0 (0.316)

### 3.4. (b) Comfort and Fatigue—Cavity Preparation (Table 4)

Mean comfort improved significantly in the palm (*p* = 0.0002), the wrist (*p* = 0.0368) and the fingers (*p* = 0.0368) when the sheath was used. Mean fatigue was also significantly less in healthy testers when the sheath was used (*p* = 0.015) (Table 4).

**Table 4:** Mean Comfort and Fatigue in dentists during cavity preparation on a scale of 0–10, where 0 is best and 10 is worst.

	No Sheath (n = 10)	Sheath (n = 10)
Mean Comfort Palm (S.D.)	1.9 (0.88)	1.1 (0.18)
Mean Comfort Wrist (S.D.)	1.9 (0.568)	1.5 (0.127)
Mean Comfort Fingers (S.D.)	1.9 (0.5676)	1.5 (0.127)
Mean Fatigue (S.D.)	1.9 (0.568)	1.4 (0.199)

Overall, all testers rated comfort and fatigue as being better when working with the sheath vs. without it. This observation was consistent in all tester groups, regardless of clinician type or MSD status.

### 3.5. Tester Open-Ended Written Comments (Table 5)

In the free comments that testers were asked to provide, the majority of testers expressed a preference for working with the ergonomic sleeve. Their comments are presented in Table 5.

**Table 5:** Testers’ Comments.

	Comfort	Fatigue	Overall Feel	Comments
No Sleeve	No comments	No comments	1/30 testers: Slightly better tactile feedback	None
With Sleeve	30/30 testers: More comfortable, due to the instrument feeling slightly cushioned, less cold, and hard.	27/30 testers: Because we didn’t need to grip so hard, we got less tired.	26/30 testers: Less hard on the fingers and hand- makes instrument feel softer and gentler and allows for better instrument control	12/30 testers: didn’t like the extra time it takes to apply and remove the sleeve
	24/30 testers: Hands need to grip less hard with sheath, so less fatigue and less slippage	21/30 testers: Hand felt less stressed after instrumentation	7/30 testers: Sleeve felt better without gloves than with them	
	20/30 testers: Instrument felt more stable in hand			

## 4. Discussion

The goal of this study was to investigate the effects on the operator’s ergonomic load of a new, soft and flexible ergonomic handle sheath for power-driven dental tools. Outcomes measures included muscle work, fatigue and comfort. These variables were evaluated using two standardized clinical tasks: targeted dental prophylaxis by dental hygienists using a piezoelectric scaler, and four specific dental cavity preparations by dentists using a steel dental micromotor. These devices were selected because they are both frequently employed in the dental office. They cause a considerable amount of vibration during use and are considered to be an important cause of and trigger for MSDs [50,51]. Because work-related MSDs are especially common in dental hygienists, the same scaling task was performed by hygienists with and without MSDs, to allow a comparison of the work expended and the fatigue experienced by healthy clinicians vs. those with MSDs. 

All clinical testers included in this study were right-handed, to ensure that any positional differences related to handedness did not affect the results [52,53]. Studies have shown that left-handed dental clinicians may be more prone to MSDs because the entirety of instrumentation and positioning involved in dental procedures has been developed for right-handed individuals [53,54]. The effect of the test sheath on the ergonomic performance of dental devices in left-handed dental clinicians who are healthy, and in those with existing MSDs is currently under evaluation.

In the first arm of this study, hygienists with and without MSDs completed a standardized scaling task using a piezoelectric ultrasonic scaler. To the best of our knowledge, this is the first study that evaluates the effect of MSDs on the amount of muscle work needed to complete a set dental task using dental motor-driven tools, and on clinician comfort and fatigue. The findings from this pilot investigation indicate that hygienists with MSDs may expend more muscle work than their healthy counterparts to complete a set scaling task. Moreover, their own perceived comfort and fatigue after piezoelectric scaling are significantly worse than in testers without MSDs. It is likely that a better understanding of how MSDs affect muscle dynamics during instrumentation may lead to new and better avenues of prevention, mitigation and treatment for instrumentation-related disorders. This becomes particularly important in the greater context of the work-health balance: researchers estimate that 33.8 to 95.3% of the entire workforce has MSDs, primarily related to the lower back, neck, upper back and shoulder [29]. 

The results of the first arm of this study also demonstrated that, when hygienists with MSDs used the piezoelectric ultrasonic scaler with the test ergonomic sheath in place, their comfort and fatigue levels improved considerably, approaching those of healthy hygienists. One explanation for this finding might be that the more comfortable handle surface provided by the ergonomic sheath might allow clinicians with MSDs to adopt healthier grip configurations and working patterns, thus avoiding or at least reducing the discomfort and greater workload associated with suboptimal grip, grasp and instrumentation trajectories. Additional studies are planned to investigate this important point. A better understanding of why clinicians with MSDs require more muscle work to complete a clinical task may pave the pathway to developing new mitigation approaches that might allow these individuals to resume some of their clinical activities. 

Using the ergonomic sheath during scaling significantly reduced muscle work in all hygienists. This effect was somewhat greater in hygienists with MSDs than in their healthy counterparts, but the difference between the two clinician groups did not reach the level of statistical significance. 

The presence of the ergonomic sheath during standardized cavity preparation by dentists also reduced the amount of overall expended muscle work. It is unclear to what extent the role of the sheath in damping handpiece vibration vs. its ergonomically beneficial effects in providing a softer, warmer handle contributed to this benefit. Thus, the results of the current study support the findings of previous research in favor of softer, warmer, non-metal dental instrument handles [34,35,39,40,43,44,55,56,57,58,59], and lend weight to the concept of expanding these concepts from hand-held instruments to motor-driven dental tools, be it as an add-on such as the removable sheath tested in this study, or as a feature integrated into the design of the device handle. Some studies outside the field of dentistry have found beneficial effects from innovative approaches that extend beyond instrument handle design to more extensive measures such as localized structural exoskeletons, targeted support and psychosocial or stress-preventive interventions [60,61,62,63]. 

In this study, there was strong agreement between the direct sEMG measurements of expended muscle work and the semi-quantitative VAS scores with which testers rated their instrumentation-related comfort and fatigue. The agreement between the two evaluation modalities was excellent in all tester groups, regardless of clinician type or MSD status. Using VAS scores, clinicians predominantly reported better comfort and less fatigue when the ergonomic sheath was in place during scaling and cavity preparation. Similarly, the corresponding sEMG data evidenced that all testers expended significantly less muscle work to complete their set task when the ergonomic sheath was in place than in its absence. These findings align well with those from previous studies, in which investigators reported that both objective and subjective measurements are needed for meaningful evaluation of ergonomic performance in dental hand instruments [57,64,65]. Such measures typically include neurophysiological evaluation of work in the specific muscles that are directly involved in a targeted activity, as well as the more subjective VAS-based measures of fatigue or comfort that were also used in the current study. 

In summary, clinicians with MSDs work harder and experience more fatigue related to completing a specific clinical task than their healthy counterparts. Moreover, a novel ergonomic handle sheath for motor-driven dental tools may improve the ergonomic performance of these devices, especially in clinicians with MSDs. 

Limitations of this study include the relatively small sample size and the age discrepancy between healthy hygienists and those with MSDs. Initially, we had attempted to achieve a similar age range in both groups, but it soon became clear during recruitment that this was not possible. Young hygienists typically do not suffer from MSDs, and MSDs develop and progress with increasing age. This limitation is mitigated by the statistically similar number of years of experience in each group of hygienists. Moreover, it would have been ideal to test the sheath in dentists with MSDs also, and this research is planned in future studies. Finally, body positioning during instrumentation may well affect work, comfort and fatigue during instrumentation, and this variable requires investigation in future studies. In this first pilot study, all testers were asked to position themselves however they felt most comfortable and according to their customary usage in order to avoid extraneous noise in the study data from the muscle work required to adopt a position foreign to them. However, some form of standardization of positioning would also provide benefits. This is a point that requires further investigation. Additional clinical studies are now underway to expand the investigational scope, sample size and duration of this research, and to evaluate the effect of the novel sheath on instrumentation efficacy and speed, tactile feedback as well as hand, wrist and body positioning during instrumentation. In these studies, the severity and duration of MSDs in testers are also evaluated in much greater detail, so that a more nuanced understanding of the relationship between injury type/location, specific clinical tasks and ergonomic outcomes can be established. 

## 5. Conclusions

The results of this pilot study indicate that a novel ergonomic sheath for dental power-driven instruments may include improved comfort, less muscle work and reduced fatigue during dental procedures, especially in individuals with MSDs. Additional studies are underway.

## Data Availability

The data are not publicly available due to their containing information that could compromise the privacy of research participants. The data that support the findings of this study are available on request from the corresponding author, [PWS].

## References

[1] De Sio S., Traversini V., Rinaldo F., Colasanti V., Buomprisco G., Perri R., Mormone F., La Torre G., Guerra F. (2018). Ergonomic risk and preventive measures of musculoskeletal disorders in the dentistry environment: An umbrella review. PeerJ.

[2] Leigh J.P., Miller T.R. (1998). Occupational illnesses within two national data sets. Int. J. Occup. Environ. Health.

[3] Suedbeck J.R., Tolle S.L., McCombs G., Walker M.L., Russell D.M. (2017). Effects of Instrument Handle Design on Dental Hygienists’ Forearm Muscle Activity During Scaling. J. Dent. Hyg..

[4] Humann P., Rowe D.J. (2015). Relationship of Musculoskeletal Disorder Pain to Patterns of Clinical Care in California Dental Hygienists. J. Dent. Hyg..

[5] Harris M.L., Sentner S.M., Doucette H.J., Brillant M.G.S. (2020). Musculoskeletal disorders among dental hygienists in Canada. Can. J. Dent. Hyg..

[6] Lalumandier J.A., McPhee S.D., Riddle S., Shulman J.D., Daigle W.W. (2000). Carpal tunnel syndrome: Effect on Army dental personnel. Mil. Med..

[7] Lalumandier J.A., McPhee S.D. (2001). Prevalence and risk factors of hand problems and carpal tunnel syndrome among dental hygienists. J. Dent. Hyg..

[8] Werner R.A., Franzblau A., Gell N., Hamann C., Rodgers P.A., Caruso T.J., Perry F., Lamb C., Beaver S., Hinkamp D. (2005). Prevalenceof upper extremity symptoms and disorders among dental and dental hygiene students. J. Calif. Dent. Assoc..

[9] Osborn J.B., Newell K.J., Rudney J.D., Stoltenberg J.L. (1990). Carpal tunnel syndrome among Minnesota dental hygienists. J. Dent. Hyg..

[10] Anton D., Rosecrance J., Merlino L., Cook T. (2002). Prevalence of musculoskeletal symptoms and carpal tunnel syndrome among dental hygienists. Am. J. Ind. Med..

[11] Marshall E.D., Duncombe L.M., Robinson R.Q., Kilbreath S.L. (1997). Musculoskeletal symptoms in New South Wales dentists. Aust. Dent. J..

[12] Osborn J.B., Newell K.J., Rudney J.D., Stoltenberg J.L. (1990). Musculoskeletal pain among Minnesota dental hygienists. J. Dent. Hyg..

[13] Åkesson I., Johnsson B., Rylander L., Moritz U., Skerfving S. (1999). Musculoskeletal disorders among female dental personnel: Clinical examination and a 5-year follow-up study of symptoms. Int. Arch. Occup. Environ. Health.

[14] Milerad E., Ekenvall L. (1990). Symptoms of the neck and upper extremities in dentists. Scand. J. Work. Environ. Health..

[15] Rundcrantz B.L. (1991). Pain and discomfort in the musculoskeletal system among dentists. Swed. Dent. J..

[16] Oberg T., Oberg U. (1993). Musculoskeletal complaints in dental hygiene: A survey study from a Swedish county. J. Dent. Hyg..

[17] Corks I. (1997). Occupational health hazards in dentistry: Musculoskeletal disorders. Ont. Dent..

[18] Fish D.R., Morris-Allen D.M. (1998). Musculoskeletal disorders in dentists. NY State Dent. J..

[19] Finsen L., Christensen H., Bakke M. (1998). Musculoskeletal disorders among dentists and variation in dental work. Appl. Ergon..

[20] Akesson I., Schütz A., Horstmann V., Skerfving S., Moritz U. (2000). Musculoskeletal symptoms among dental personnel-lack of association with mercury and selenium status, overweight and smoking. Swed. Dental J..

[21] Alexopoulos E.C., Stathi I., Charizani F. (2004). Prevalence of musculoskeletal disorders in dentists. BMC Musculoskelet. Disord..

[22] Auguston T.E., Morken T. (1996). Musculoskeletal problems among dental health personnel. A survey of the public dental health services in Hordaland. Tdsskr Nor. Laegeforen..

[23] Chowanadisai S., Kukiattrakoon B., Yapong B., Kedjarune U., Leggat P.A. (2000). Occupational health problems of dentists in southern Thailand. Int. Dent. J..

[24] Taib M.F.M., Bahn S., Yun M.H., Taib M.S.M. (2017). The effects of physical and psychosocial factors and ergonomic conditions on the prevalence of musculoskeletal disorders among dentists in Malaysia. Work..

[25] Ratzon N.Z., Yaros T., Mizlik A., Kanner T. (2000). Musculoskeletal symptoms among dentists in relation to work posture. Work..

[26] Rundcrantz B., Johnsson B., Moritz U. (1990). Cervical pain and discomfort among dentists. Epidemiological, clinical and therapeutic aspects. Swed. Dent. J..

[27] Al-Huthaifi B.H., Al Moaleem M.M., Alwadai G.S., Nassar J.A., Sahli A.A.A., Khawaji A.H., Juraybi A.K., Alsheri Y.A., Aldhorae K., Yaqoub A.A. (2023). High Prevalence of Musculoskeletal Disorders Among Dental Professionals: A Study on Ergonomics and Workload in Yemen. Med. Sci. Monit..

[28] Ćwirzeń W., Wagner L. (2023). Evaluating the Dental Hygienists’ Exposure to the Risk of Musculoskeletal Disorders. Eur. J. Dent..

[29] Kumar M., Pai K.M., Vineetha R. (2020). Occupation-related musculoskeletal disorders among dental professionals. Med. Pharm. Rep..

[30] Marklund S., Mienna C.S., Wahlström J., Englund E., Wiesinger B. (2020). Work ability and productivity among dentists: Associations with musculoskeletal pain, stress, and sleep. Int. Arch. Occup. Environ. Health.

[31] Valachi B. (2009). Practice Dentistry Pain-free: Evidence-based Strategies to Prevent Pain and Extend Your Career. Br. Dent. J..

[32] Schlenker A., Kapitán M., Vavřičková L., Bušová M. (2020). Assessment of local muscular load of dental practitioners. Cent. Eur. J. Public. Health.

[33] Åkesson I., Hansson G., Balogh I., Moritz U., Skerfving S. (1997). Quantifying workload in neck, shoulders and wrists in female dentists. Int. Arch. Occup. Environ. Health.

[34] Dong H., Barr A., Loomer P., Rempel D. (2005). The effects of finger rest positions on hand muscle load and pinch force in simulated dental hygiene work. J. Dent. Educ..

[35] Dong H., Loomer P., Barr A., LaRoche C., Young E., Rempel D. (2007). The effect of tool handle shape on hand muscle load and pinch force in a simulated dental scaling task. Appl. Ergon..

[36] Jonker D., Rolander B., Balogh I. (2009). Relation between perceived and measured workload obtained by long-term inclinometry among dentists. Appl. Ergon..

[37] Finsen L. (1999). Biomechanical aspects of occupational neck postures during dental work. Int. J. Ind. Ergon..

[38] Wink C., Yang S.M., Habib A.A., Lin K., Takesh T., Wilder-Smith P. (2024). Effect of a Novel Adaptive Handle Design on the Ergonomic Performance of Periodontal Curettes in Dental Hygienists with and without Musculoskeletal Disorders: A Pilot Clinical Study. Dent. J..

[39] Wilkins E.M. (1999). Instruments and Principles for Instrumentation. Clinical Practice of Dental Hygienist..

[40] Gehrig J.S., Sroda R., Saccuzzo D. (2017). Ergonomic Risk Factors Associated with Periodontal Instrumentation. Fundamentals of Periodontal Instrumentation & Advanced Root Instrumentation.

[41] McCombs G., Russell D.M. (2014). Comparison of Corded and Cordless Handpieces on Forearm Muscle Activity, Procedure Time and Ease of Use during Simulated Tooth Polishing. J. Dent. Hyg..

[42] Enders L.R., Seo N.J. (2011). Phalanx force magnitude and trajectory deviation increased during power grip with an increased coefficient of friction at the hand-object interface. J. Biomech..

[43] Lin K., Wink C., Dolan B., Osann K., Habib A.A., Gehrig J., Wilder-Smith P. (2023). A Novel Ergonomic Curette Design Reduces Dental Prophylaxis-Induced Muscle Work and Fatigue. Dent. J..

[44] Das D., Strasser H. (2007). Ergonomic evaluation, design and testing of hand tools. Assessment of the Ergonomic Quality of Hand-Held Tools and Computer Input Devices.

[45] Jarvik J.G., Yuen E., Kliot M. (2004). Diagnosis of carpal tunnel syndrome: Electrodiagnostic and MR imaging evaluation. Neuroimag Clin. N. Am..

[46] Burden A., Bartlett R. (1999). Normalisation of EMG amplitude: An evaluation and comparison of old and new methods. Med. Eng. Phys..

[47] Bolga L.A., Uhl T.L. (2007). Reliability of electromyographic normalization methods for evaluating the HIP musculature. J. Electromyogr. Kinesiol..

[48] Netto K.J., Burnett A.F. (2006). Reliability of normalization methods for EMG analysis of neck muscles. Work..

[49] Gemne G., Saraste H. (1987). Bone and joint pathology in workers using hand-held vibrating tools. An overview. Scand. J. Work. Environ. Health.

[50] Morse T.F., Michalak-Turcotte C., Atwood-Sanders M., Warren N., Peterson D.R., Bruneau H., Cherniack M. (2003). A pilot study of hand and arm musculoskeletal disorders in dental hygiene students. J. Dent. Hyg..

[51] Ortiz Simon J.L., Martinez A.M., Espinoza D.L., Romero Velazquez J.G. (2011). Mechatronic assistant system for dental drill handling. Int. J. Med. Robot..

[52] Kaya M.D., Orbak R. (2004). Performance of left-handed dental students is improved when working from the left side of the patient. Int. J. Ind. Ergon..

[53] Tezel A., Kavrut F., Tezel A., Kara C., Demir T., Kavrut R. (2005). Musculoskeletal disorders in left- and right-handed Turkish dental students. Int. J. Neurosci..

[54] Simmer-Beck M., Branson B.G. (2010). An evidence-based review of ergonomic features of dental hygiene instruments. Work..

[55] Hayes M.J. (2017). The Effect of Stainless Steel and Silicone Instruments on Hand Comfort and Strength: A pilot study. J. Dent. Hyg..

[56] Dianat I., Nedaei M., Nezami M. (2015). The effects of tool handle shape on hand performance, usability and discomfort using masons’ trowels. Int. J. Ind. Ergon..

[57] Nevala N. (2013). Evaluation of Ergonomics and Efficacy of Instruments in Dentistry. Ergon. Open J..

[58] Rempel D., Lee D.L., Dawson K., Loomer P. (2012). The effects of periodontal curette handle weight and diameter on arm pain: A four-month randomized controlled trial. J. Am. Dent. Assoc..

[59] Dong H., Barr A., Loomer P., LaRoche C., Young E., Rempel D. (2006). The effects of periodontal instrument handle design on hand muscle load and pinch force. J. Am. Dent. Assoc..

[60] Pinho J.P., Parik Americano P., Taira C., Pereira W., Caparroz E., Forner-Cordero A. (2020). Shoulder muscles electromyographic responses in automotive workers wearing a commercial exoskeleton. Annu. Int. Conf. IEEE Eng. Med. Biol. Soc..

[61] Pinho J.P., Forner-Cordero A. (2022). Shoulder muscle activity and perceived comfort of industry workers using a commercial upper limb exoskeleton for simulated tasks. Appl. Ergon..

[62] Pacifico I., Aprigliano F., Parri A., Cannillo G., Melandri I., Sabatini A.M., Violante F.S., Molteni F., Giovacchini F., Vitiello N. (2023). Evaluation of a spring-loaded upper-limb exoskeleton in cleaning activities. Appl. Ergon..

[63] Deeney C., O’Sullivan L. (2009). Work related psychosocial risks and musculoskeletal disorders: Potential risk factors, causation and evaluation methods. Work.

[64] Strasser H., Wang B., Hoffmann A. (1996). Electromyographic and subjective evaluation of hand tools: The example of masons’ trowels. Int. J. Ind. Ergon..

[65] Kuijt-Evers L., Bosch T., Huysmans M., de Looze M., Vink P. (2007). Association between objective and subjective measurements of comfort and discomfort in hand tools. Appl. Ergon..

